# Pathogenicity and Genomic Characteristics Analysis of *Pasteurella multocida* Serotype A Isolated from Argali Hybrid Sheep

**DOI:** 10.3390/microorganisms12061072

**Published:** 2024-05-25

**Authors:** Xinyan Cao, Lanying Gu, Zhiyu Gao, Wenyu Fan, Qinchuan Zhang, Jinliang Sheng, Yanbing Zhang, Yanming Sun

**Affiliations:** College of Animal Science and Technology, Shihezi University, Shihezi 832003, China; caoxinyan12@outlook.com (X.C.); gulanying6688@163.com (L.G.); gaozhiyu1234@live.com (Z.G.); fanwenyu@email.swu.edu.cn (W.F.); qinchuanzhang2020@163.com (Q.Z.); shengjinliang@shzu.edu.cn (J.S.)

**Keywords:** argali hybrid sheep, *Pasteurella multocida*, whole genome sequencing, pathogenicity

## Abstract

Respiratory diseases arising from co-infections involving *Pasteurella multocida* (*P. multocida*) and *Mycoplasma ovipneumoniae* (Mo) pose a substantial threat to the sheep industry. This study focuses on the isolation and identification of the *P. multocida* strain extracted from the lung tissue of an argali hybrid sheep infected with Mo. Kunming mice were used as a model to assess the pathogenicity of *P. multocida*. Subsequently, whole genome sequencing (WGS) of *P. multocida* was conducted using the Illumina NovaSeq PE150 platform. The whole genome sequencing analysis involved the construction of an evolutionary tree to depict conserved genes and the generation of a genome circle diagram. *P. multocida,* identified as serotype A, was named *P. multocida* SHZ01. Our findings reveal that *P. multocida* SHZ01 infection induces pathological manifestations, including hemorrhage and edema, in mice. The phylogenetic tree of conserved genes analyzing *P. multocida* from different countries and different host sources indicates close relatedness between the *P. multocida* SHZ01 strain and the *P. multocida* 40540 strain (A:12), originating from turkeys in Denmark. The genome of *P. multocida* SHZ01 comprises 2,378,508 base pairs (bp) with a GC content of 40.89%. Notably, this strain, designated *P. multocida*, exhibits two distinct gene islands and harbors a total of 80 effector proteins associated with the Type III Secretion System (T3SS). The *P. multocida* SHZ01 strain harbors 82 virulence genes and 54 resistance genes. In the *P. multocida* SHZ01 strain, the proteins, genes, and related GO and KEGG pathways have been annotated. Exploring the relationship between these annotations and the pathogenicity of the *P. multocida* SHZ01 strain would be valuable. This study holds great significance in further understanding the pathogenesis and genetic characteristics of the sheep-derived *P. multocida* SHZ01 strain. Additionally, it contributes to our understanding of respiratory diseases in the context of co-infection.

## 1. Introduction

*P. multocida* can infect a variety of livestock, as well as wild animals, humans, and nonhuman primates, causing sheep pneumonia, fowl cholera, progressive atrophic rhinitis in swine, and lower respiratory tract infections in sheep and pigs. Its impact on sheep, leading to severe respiratory diseases, has implications for the intensive development of sheep farming in our country [1]. The current classification system separates *P. multocida* into five capsule serotypes (A, B, D, E, and F) and sixteen lipopolysaccharide antigen serotypes [2]. Certain serotypes exhibit distinct pathogenicities in different animal species. For instance, fatal pneumonia from sheep, goat and cattle, and avian cholera is primarily attributed to serotype A, while hemorrhagic sepsis, causing acute disease in cattle and buffaloes, is associated with serotypes B and E. This condition is characterized by edematous swelling of the head and neck, coupled with hemorrhagic lymphadenopathy. Additionally, the *P. multocida* of serotype D expressing *P. multocida* toxin (PMT) has been identified as a causative agent of atrophic rhinitis in pigs. The pathogenicity of *P. multocida* has a series of virulence factors, including genes encoding capsule formation, lipopolysaccharide (LPS), fimbriae and other adhesins, toxins, iron-regulated iron acquisition proteins, sialic acid metabolism, hyaluronidase, and outer membrane proteins (OMPs) [3]. In the process of studying the genotyping of *P. multocida* infection in sheep abroad, capsular serotypes A and D were mainly isolated from sheep lungs and goat lungs [4]. However, *P. multocida* of serotype B predominantly affects sheep in China, while serotypes A and D have been isolated in limited geographical areas [5].

In recent years, secondary bacterial infections, particularly those caused by *Mycoplasma ovipneumoniae* (Mo), have emerged as a significant threat to animal health. Mixed infections involving *P. multocida* and Mo are frequently observed in sheep flocks, with reports indicating a high prevalence of double-positive antibodies for Mo and *P. multocida*, reaching up to 83% in sheep flocks in Xinjiang and other regions [6]. International scholars have suggested a potential association between mixed infections of mycoplasma and bacteria, particularly those involving Mo and *P. multocida*, and the occurrence of argali pneumonia [7]. In the nasal swab samples of argali sheep from Wyoming, USA, the positive rate of Mo detection was 85.7%. The detection rate of Mo in the lung tissue of argali sheep after death was 57.1%, and the detection rate of *P. multocida* was 100% [8]. The mixed infection of Mo and *P. multocida* is the main factor leading to the death of wild argali sheep [9]. Respiratory diseases in sheep and goats are extensively documented globally, with *P. multocida* serotypes recognized as significant bacterial pathogens. This underscores the global importance of *P. multocida* in respiratory health, emphasizing the need for a comprehensive understanding of serotype-specific effects [10].

Whole genome sequencing (WGS) is a powerful genetic technique, employed to unravel the intricate pathogenic mechanisms of bacteria under specific pathological conditions [11]. The current landscape of WGS includes second-generation sequencing, such as Illumina technology, and third-generation sequencing, exemplified by single-molecule real-time (SMRT) sequencing [12]. These methods allow for the quick and cost-effective sequencing of the entire genome of an organism. Despite the widespread application of WGS in deciphering the genomic makeup of *P. multocida* in various animal species, the complete genomic information of *P. multocida* in argali hybrid sheep remains elusive. The difference between *P. multocida* in argali hybrid sheep and other strains from different hosts in terms of the whole genome characteristics and pathogenicity pathway is poorly understood.

In this study, Kunming mice were used as a model to assess the pathogenicity of the *P. multocida* SHZ01 strain. The SHZ01 strain was isolated from the lungs of argali hybrid sheep infected with Mo. Additionally, an analysis of the strain’s whole genome sequencing data was conducted to unravel its genetic composition and attributes. The outcomes of our investigation have revealed a wealth of information within the genome sequence of the *P. multocida* SHZ01 strain. Numerous genes associated with virulence and drug resistance were predicted, shedding light on the molecular foundations that could be leveraged for the prevention and treatment of *P. multocida* infection in sheep. These findings contribute to the expanding knowledge of *P. multocida*’s pathogenicity and provide potential avenues for the targeted treatment of respiratory diseases in sheep.

## 2. Materials and Methods

### 2.1. Statement on Experimental Animals and Ethics

Argali hybrid sheep were used to collect samples. An ethical declaration was made with the sheep owners in accordance with the guidelines for the care and use of research animals. The owner’s consent was obtained for research performed on the animal. Twelve female healthy Kunming mice, aged 6–8 weeks, were employed in the assessment of the pathogenicity of the *P. multocida* SHZ01 strain. All animals were housed individually in designated cages within the animal facility at Shihezi University throughout the experiment, ensuring controlled and monitored conditions. The animal experiments that were conducted adhered to ethical standards and were approved by the Animal Experiment Ethics Committee, with approval reference A2023-017. This committee ensures that all experimental procedures involving animals comply with ethical guidelines and prioritize the welfare of the animals used in scientific research.

### 2.2. Sample Preparation

Argali hybrid sheep were obtained from a small breeding enterprise in 170th Regiment of the 9th Division, Production and Construction Corps, Xinjiang Tacheng area, China [13]. All sheep were housed in appropriate pens and fed without antibiotics. Samples were obtained from an argali hybrid sheep only infected with the Mo Y98 strain. The sheep infected with Mo developed clinical symptoms of pneumonia: coughing and runny nose. The sheep were humanely euthanized using sodium pentobarbital and sacrificed by exsanguination from the carotid artery. Subsequently, the intact lung was carefully removed and the trachea was ligated. A pre-cooled phosphate buffer solution (PBS), sterilized in advance at 4 °C, was prepared. Using aseptic techniques, pre-cooled 1× PBS was poured into the lungs, which were gently massaged, and the lavage fluid was collected in a sterile Erlenmeyer flask. Lavage fluid was centrifuged at 12,000 revolutions per minute (rpm) and 10 °C for 8 min, and centrifugal precipitation was collected.

### 2.3. Bacterial Isolation and Identification

To facilitate bacterial culture, Trypticase Soy Agar (TSA) solid medium and Trypticase Soy Broth (TSB) liquid medium were prepared beforehand. The collected centrifugal precipitation of cell lavage fluid was then inoculated onto TSA plates using an inoculation loop within a clean bench. The TSA plates were inverted and subsequently incubated overnight in a constant-temperature incubator set at 37 °C. Following this, the incubation, isolation, and purification of *P. multocida* were carried out. Individual colonies were selected and inoculated into TSB culture medium. After culturing for 4–6 h at 37 °C with shaking at 200 rpm, 16S rRNA identification was performed. Upon the alignment of comparison results with the target criteria, primers were designed based on the references provided by Townsend, et al. [14] (Table S1). Subsequently, bacterial DNA was extracted using the TIANamp Bacterial DNA Kit. Polymerase chain reaction (PCR) was used to identify bacterial species. The extracted DNA served as a template for PCR assay, as detailed in Table S2. Detailed PCR reaction procedures are shown in Table S3.

### 2.4. Capsular Serological Identification Is Performed

The capsular serotype primer sequences of *P. multocida* (KMT1, hyaD-hyaC, bcbD, dcbF, ecbJ, fcbD) were determined through reference to the literature [14], and the primers were synthesized at Sangon Biotech (Shanghai) Co., Ltd. in China, as detailed in Table S1. Synthetic primers were used to identify the serotype of the isolated *P. multocida*, and the PCR reaction procedures are shown in Table S4.

### 2.5. Assessment of Mouse Pathogenicity

*P. multocida* stored in glycerol was revived in Trypticase Soy Broth (TSB) medium and cultured until the optical density (OD) value reached approximately 0.6. The concentration of *P. multocida* was measured using the gradient dilution method (10, 10^2^, 10^3^, 10^4^, 10^5^, 10^6^, 10^7^, 10^8^). According to a previous study [10], the measured concentration was then adjusted to 1.58 × 10^6^ colony-forming units (CFU). For the in vivo assessment of pathogenicity, twelve female Kunming mice were randomly divided into experimental and control groups (n = 6, each), with the former receiving an abdominal cavity injection of the *P. multocida* culture (1.58 × 10^6^ CFU) and the latter injected with sterilized PBS. Each group of mice were kept in separate cages. Throughout the experiment, systematic monitoring and documentation of the mice’s feeding situation, mental state, and mortality were conducted at regular intervals. Dissection of deceased mice facilitated the observation of pathological changes in the lungs, spleen, and liver. After 24 h, surviving mice were euthanized by CO_2_ asphyxiation, followed by cervical dislocation according to the “Guidelines on the Humane Treatment of Laboratory Animals” (policy no. A2023-017). Lung tissue with evident lesions was aseptically isolated for bacteria identification, while the remaining lung tissue was fixed and subjected to hematoxylin and eosin (HE) staining. An biological microscope (the brand of soptop; model EX30) was utilized for examination, and lung injury scores were determined using the Smith method [15].

### 2.6. Library Construction and Genome Sequencing 

The whole genome sequencing process was conducted by Novogene (Beijing) Co., Ltd. in China, employing a rigorous workflow. Genomic DNA extraction utilized either the SDS or STE method, with subsequent assessment of purity and integrity through agarose gel electrophoresis. Precise quantification of DNA was achieved using the Qubit^®^ 2.0 Fluorometer (Thermo Fisher Scientific, Waltham, MA, USA). DNA fragments underwent controlled random shearing, resulting in fragments approximately 350 bp in length. The NEBNext^®^ Ultra™ DNA Library Prep Kit for Illumina (NEB, Ipswich, MA, USA) facilitated comprehensive library preparation, encompassing essential steps such as end repair, A-tail addition, sequencing adapter addition, purification, PCR amplification, and more. Post-library construction, the initial quantification using Qubit 2.0 preceded library dilution to a concentration of 2 ng/μL. Agilent 2100 (Santa Clara, CA, USA) was employed to detect the insert fragment, and the Q-PCR method precisely determined the effective library concentration. Stringent quality inspection criteria were met before subjecting the libraries to sequencing on the Illumina NovaSeq PE150 platform (San Diego, CA, USA). This integrated approach ensures the generation of high-quality genomic data for subsequent in-depth analysis and interpretation, adhering to industry standards and best practices in whole genome sequencing.

### 2.7. Genome Assembly

The preliminary assembly of the clean data, following quality control, is undertaken using SOAP denovo (version 2.04), SPAdes (http://bioinf.spbau.ru/spades, accessed on 20 April 2024), and ABySS assembly (http://www.bcgsc.ca/platform/bioinfo/software/abyss, accessed on 20 April 2024) software. To enhance the assembly results by optimizing and filling gaps, the gapclose software (Version: 1.12) is employed. The final assembly result is obtained after integration using CISA software (http://sb.nhri.org.tw/CISA/, accessed on 20 April 2024). Fragments with a length below 500 bp are filtered out, and contaminated samples undergo a decontamination process. Subsequent to the assembly, gene prediction is conducted, and the results are subject to a thorough evaluation and statistical analysis, ensuring the robustness and accuracy of the final genomic assembly. This systematic approach to assembly and analysis aligns with established practices in genomics, incorporating a range of software tools to ensure the fidelity and completeness of the genomic data.

### 2.8. Analysis of Genome Components

For the prediction and annotation of genomic elements, a suite of bioinformatics tools was systematically employed. GeneMarkS software (Version 4.17) played a pivotal role in accurately predicting coding genes within the genomic sequence [16]. To identify and mask dispersed repeat sequences, RepeatMasker software (Version open-4.0.5) was applied [17]. Tandem repeat sequences were discerned through the utilization of TRF (Tandem Repeats Finder, Version 4.07b) software [18]. For the prediction of transfer RNA (tRNA), ribosomal RNA (rRNA), and small RNA (sRNA), the tRNAscan-SE software (Version 1.3.1) [19], rRNAmmer software (Version 1.2) [20], and the cmsearch program (Version 1.1rc4) were employed, respectively. The prediction of gene islands was accomplished using Island Path-DIOMB software (Version 0.2) [21]. Prophages within the genomic sequence were predicted using the phiSpy software (Version 2.3) [22]. The CRISPRdigger software (Version 1.0) was utilized for the prediction of clustered, regularly interspaced, short palindromic repeats (CRISPR) within the sample genome [23]. This comprehensive array of bioinformatics tools ensures a thorough and accurate annotation of various genomic features, contributing to a comprehensive understanding of the genetic landscape of the sequenced sample.

### 2.9. Analysis of Genome Functionality

The annotation of gene functions involved a comprehensive analysis using various databases, including Gene Ontology (GO), Kyoto Encyclopedia of Genes and Genomes (KEGG), Cluster of Orthologous Groups of proteins (COG), Non-Redundant Protein Database (NR), Transporter Classification Database (TCDB), Carbohydrate-Active enZYmes Database (CAZy), Pfam, and Swiss-Prot. Protein sequences of predicted genes underwent a Diamond comparison against each functional database, employing a significance threshold of evalue ≤ 1 × 10^−5^. For each sequence, the comparison results with the highest score (default identity ≥ 40%, coverage ≥ 40%) were selected for annotation. Further analyses were conducted to predict specific functionalities. The T3SS effector proteins were predicted using EffectiveT3 software (Version 1.0.1) [24], while the secondary metabolism gene cluster in the genome was identified using the antiSMASH-4.0.2 program [25]. The prediction of virulence genes and drug resistance genes was performed utilizing the Pathogen–Host Interactions Database (PHI), Virulence Factors of Pathogenic Bacteria (VFDB), Antibiotic Resistance Genes Database (ARDB), and Comprehensive Antibiotic Research Database (CARD).

From the whole genome sequencing results of the *P. multocida* SHZ01 strain, conserved gene sequences, including two 16S rRNA, three 23S rRNA, and three 50S rRNA, were selected. A BLAST algorithm of the National Center for Biotechnology Information (NCBI) platform was used for genetic sequence analysis. Fourteen distinct strains were chosen for further analysis, and the evolutionary relationships among these strains were determined by constructing an evolutionary tree using the Neighbor-Joining method implemented in MEGA 7.0.26 software.

## 3. Results

### 3.1. Identification of P. multocida Strains

**Figure 1:**
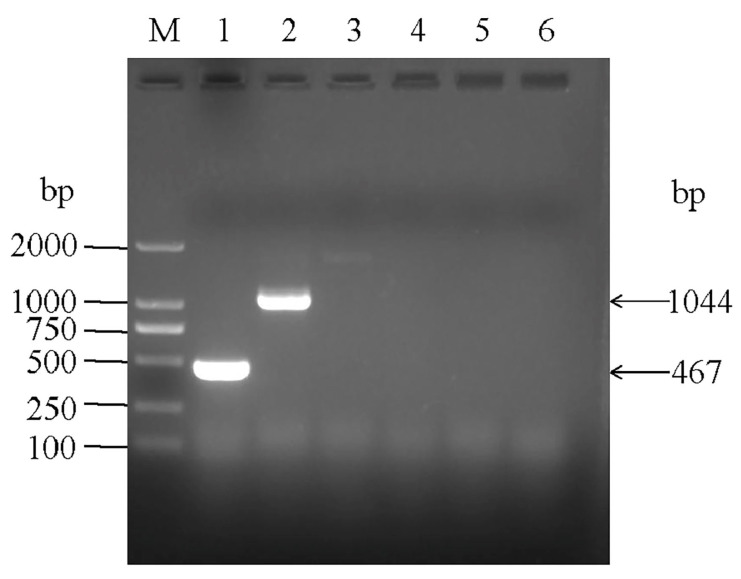
The identification of *P. multocida* SHZ01 through PCR analysis involved the amplification of specific genetic markers. The DNA fragments obtained were analyzed using a DNA marker. The specific genes targeted for amplification included Kmt1 (Lane 1), hyaD-hyaC (Lane 2), bcbD (Lane 3), dcbF (Lane 4), ecbJ (Lane 5), and fcbD (Lane 6).

The results of agarose gel electrophoresis showed that the *P. multocida*-specific primer KMT1 was used to amplify a bright band of 467 bp in size, which was consistent with the expected size of *P. multocida*, indicating that the strain was *P. multocida* and was named *P. multocida* SHZ01. Five capsular serotype primers were used to identify the serotype of the strain. The results showed that the hyaD-hyaC primers amplified a bright band of 1044 bp, which was consistent with the size of serotype A *P. multocida*, indicating that the strain was serotype A *P. multocida* (Figure 1)*. P. multocida* could be detected in four argali hybrid sheep infected with Mo (Figure S1A).

### 3.2. Mouse Lethality Assay

**Figure 2:**
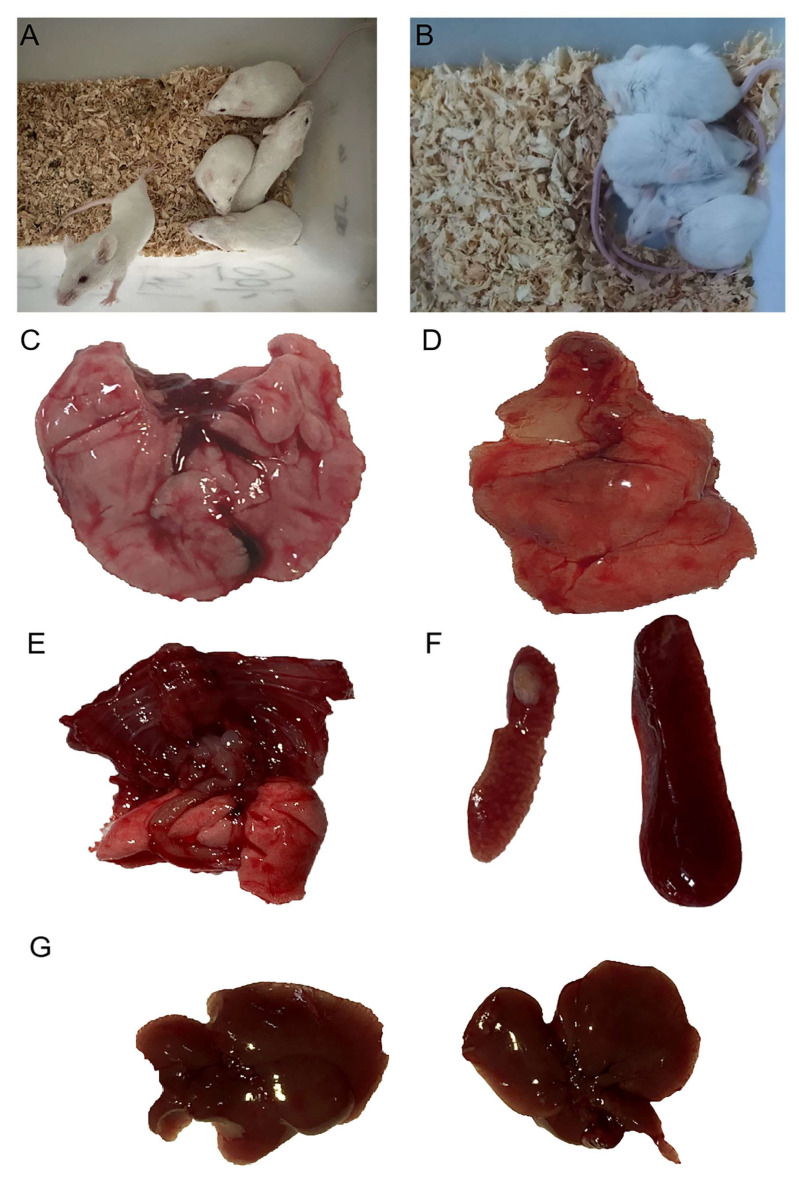
Depicts the pathological changes observed in mice following challenge with the *P. multocida* serogroup A isolate, namely, *P. multocida* SHZ01. (**A**) represents mice in the control group, while (**B**) illustrates mice in the experimental group. (**C**) displays the lung morphology of mice in the control group, showcasing a normal appearance. In contrast, (**D**,**E**) show the lungs of mice in the experimental group, revealing congestion, edema, and pulmonary adhesions. Furthermore, (**F**,**G**) illustrate the spleen and liver, respectively, in both the control and experimental groups. (**F**) demonstrates the spleen in the control group, while (**G**) showcases the enlarged spleen and liver, with small white spots on the surface, in the experimental group. These pathological findings provide visual insights into the impact of *P. multocida* SHZ01 infection on the respiratory and systemic organs of the experimental mice.

In order to investigate the virulence of the *P. multocida* SHZ01 strain, mice were challenged with *P. multocida* SHZ01 strain through an infectious dose (1.58 × 10^6^ CFU). Twenty hours post-inoculation with the *P. multocida* SHZ01 strain, distinct physiological responses were observed in the experimental and control groups of mice. The control group exhibited a regular appetite (Figure 2A), while mice inoculated with the SHZ01 strain displayed evident symptoms, including disheveled fur, depressed behavior, and aversion to cold (Figure 2B). Unfortunately, one mouse succumbed to the infection. Subsequent dissection at the 24 h mark revealed notable differences in organ morphology between the control and infected mice. The lungs of control mice appeared normal (Figure 2C), contrasting with the infected mice, who exhibited congested and edematous lungs, along with pulmonary adhesions (Figure 2D,E). Additionally, the spleen showed signs of enlargement (Figure 2F), and the liver displayed enlargement with small white spots on the surface (Figure 2G; white arrow in Figure S2). Further investigation involved the collection of lung and spleen tissues from both the deceased and control mice. Bacterial liquid PCR identification confirmed the infection in all mice, as indicated by consistent electrophoresis band sizes (Figure S1B).

**Figure 3:**
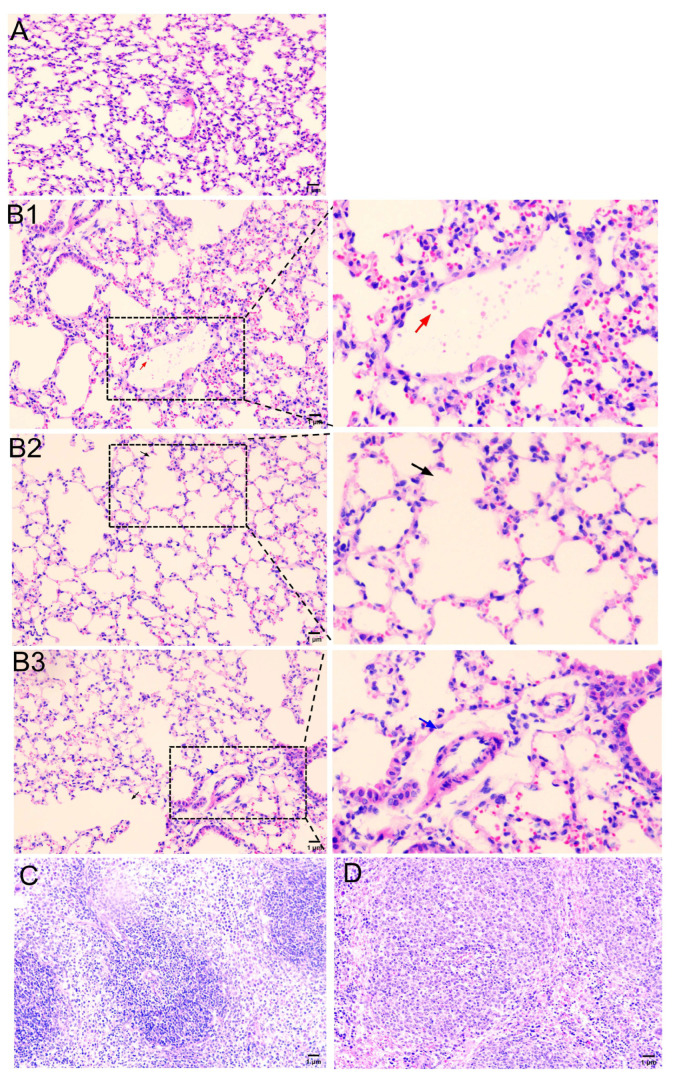
Presents histopathological observations (200×) of mice challenged with the *P. multocida* serogroup A isolate, *P. multocida* SHZ01. (**A**) illustrates the lungs of healthy mice, providing a baseline for normal lung histology. In contrast, (**B1**–**B3**) depict the lungs of mice infected with *P. multocida* SHZ01, revealing distinct histopathological changes. Specifically, (**B1**) highlights an intra-alveolar hemorrhage (red arrow), (**B2**) showcases the enlarged alveolar septa (black arrow), and (**B3**) indicates the presence of fibrinous exudation (blue arrow). (**C**) provides a reference by showing the spleen of healthy mice, demonstrating normal histological features. In contrast, (**D**) displays the spleen of mice infected with *P. multocida* SHZ01, revealing notable histopathological changes. These histopathological findings offer a detailed insight into the lung and spleen damage inflicted by the *P. multocida* SHZ01 strain, emphasizing the impact on the pulmonary microarchitecture. These histopathological observations contribute to a comprehensive understanding of the systemic effects of the bacterial challenge on the immune organ.

A pathological examination of the experimental group’s lung tissue revealed enlarged alveolar septa, intra-alveolar hemorrhage, and fibrinous exudation (Figure 3B1–B3). The spleen exhibited edema with irregularly arranged splenic cords (Figure 3D). In contrast, no apparent abnormalities were observed in the lung and spleen tissues of the control group mice (Figure 3A,C). A quantitative assessment of lung injury, using the Smith score method, demonstrated a significant increase in the degree of lung injury in mice infected with the *P. multocida* SHZ01 strain compared to the control group (3.9 vs. 0.3, *p* < 0.01). These findings indicate a moderate level of damage, with potential implications for lung function.

### 3.3. Assembly Progress and Overview of the Genome

**Figure 4:**
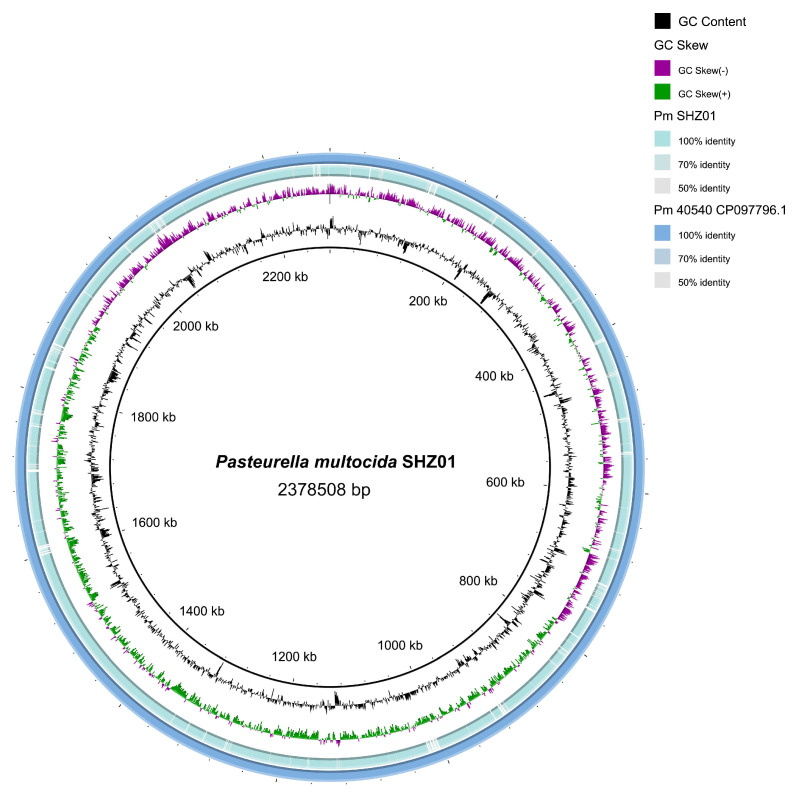
Comparative circular genome (BLAST) visualization of *P. multocida* strains. Each circle provides distinct genomic information (from outside to inside): Circles 1 and 2 display each open reading frame present in the reference genome; the compared genome is represented as an “empty” space. Circles 3, 4, and 5 illustrate GC skew+ (indicating leading strand bias), GC skew− (indicating lagging strand bias), and the GC content for the SHZ01 genome. This image was generated by BRIG.

The *P. multocida* SHZ01 strain underwent comprehensive whole genome sequencing using the advanced Illumina NovaSeq PE150 sequencing platform. The sequencing effort resulted in the assembly of 63 contigs, with the longest contig reaching a length of 173,201 base pairs. Post-assembly analysis revealed a GC content of 40.17%, while the GC content specifically associated with coding genes was determined to be 40.89%. A comparative circular genome (BLAST) visualization of the genomes of strains SHZ01 and 40540 (using the strain 40540 as a reference) is shown in Figure 4. The complete gene sequence of the *P. multocida* SHZ01 strain spans a substantial length of 2,378,508 base pairs, encompassing a total of 2418 coding genes (Table 1). To facilitate broader access to the genomic information, the entire dataset for the strain has been deposited in the National Center for Biotechnology Information (NCBI) under the unique accession number JAWWTW01000000. These sequencing and assembly details provide a foundational resource for further genomic and functional analyses of the *P. multocida* SHZ01 strain. 

**Figure 5:**
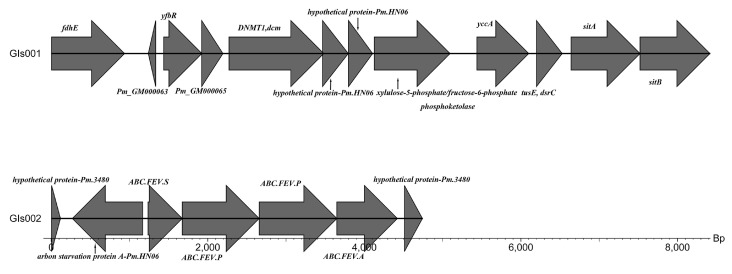
Illustrates the gene island annotation results within the genome of *P. multocida* SHZ01. The SHZ01 strain exhibits the presence of two distinct gene islands, denoted as GIs001 and GIs002. The annotated genes depicted in the figure represent the specific distribution of genes within the gene islands of the SHZ01 strain. This visualization provides a detailed overview of the genetic content and organization associated with these gene islands, shedding light on their potential functional aspects and the genomic landscape of *P. multocida* SHZ01.

Gene islands (GIs) are characterized by segments of the genome integrated through lateral gene transfer. Accessory genes clustered as a GI are desirable for the pathogenesis and adaptation of the bacteria in the host [26]. The genome of *P. multocida* SHZ01 strain was predicted to contain two GIs. *P. multocida* HN07 [27] of pig and *P. multocida* HN02 of sheep contain nine GIs and five GIs, respectively. Two GIs were harbored in the *P. multocida* SHZ01 strain, GIs001 and GIs002, as depicted in Figure 5. Moreover, the GIs001 contained two virulence-associated genes, *sitA* and *sitB*, which are related to the iron transport system. The GIs002 contained three iron transport system-associated genes: *ABC.FEV.S*, *ABC.FEV.P*, and *ABC.FEV.A*.

**Figure 6:**
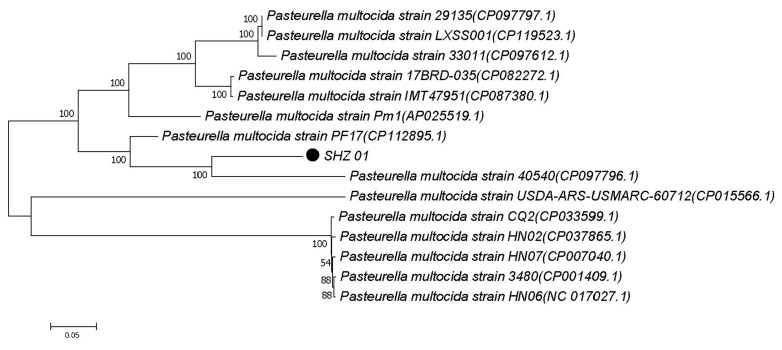
Depicts the conservative gene phylogenetic tree of *P. multocida* SHZ01. The tree is drawn to scale, with branch lengths measured in the same units as the evolutionary distances used for phylogenetic inference. The phylogenetic tree was constructed using the Neighbor-Joining method, and the computed evolutionary distances were based on the p-distance method. This phylogenetic tree provides a visual representation of the genetic relationships among different *P. multocida* strains, showcasing the position of the SHZ01 strain within the broader evolutionary context. The scale and methodology employed in constructing the tree contribute to a nuanced understanding of the evolutionary distances between the strains included in the analysis.

To elucidate the genetic relationships of conserved genes in the SHZ01 strain, an evolutionary tree was constructed, incorporating other *P. multocida* strains isolated from diverse regions and hosts (Table 2). The analysis revealed that the SHZ01 strain shares its closest genetic affinity with the *P. multocida* 40540 strain, clustering together on the same branch. Notably, the SHZ01 strain demonstrates a more recent common ancestry with the *P. multocida* PF17 strain, derived from rabbits. In contrast, substantial genetic divergence is observed between the SHZ01 strain and the *P. multocida* HN06 and 3480 strains, originating from pigs (Figure 6). Remarkably, the *P. multocida* 40540 strain, originating from turkeys and classified as serotype A:12, shares serotype similarity with the identified *P. multocida* SHZ01 strain. This phylogenetic analysis provides insights into the evolutionary relationships and genetic connections of the *P. multocida* SHZ01 strain, highlighting its distinct lineage within the broader context of *P. multocida* diversity.

### 3.4. Functional Annotation of the P. multocida Genome

To elucidate the functional aspects of the *P. multocida* SHZ01 strain’s genome, a comprehensive annotation was conducted using established databases including GO, KEGG, COG, NR, TCDB, CAZy, Pfam, Swiss-Prot, CARD, and ARDB. The majority of annotations were found in the NR, KEGG, COG, Swiss-Prot, GO, and Pfam databases, constituting 18.43%, 17.59%, 14.86%, 13.55%, 13.38%, and 13.38% of the total annotated genes, respectively (Table S5). However, the CARD database exhibited annotations for a smaller fraction, representing only 0.52% of the total gene count. Notably, no gene annotations were identified within the ARDB database, underscoring the unique genomic characteristics of the *P. multocida* SHZ01 strain.

#### 3.4.1. Annotation Outcomes for Gene Ontology (GO)

**Figure 7:**
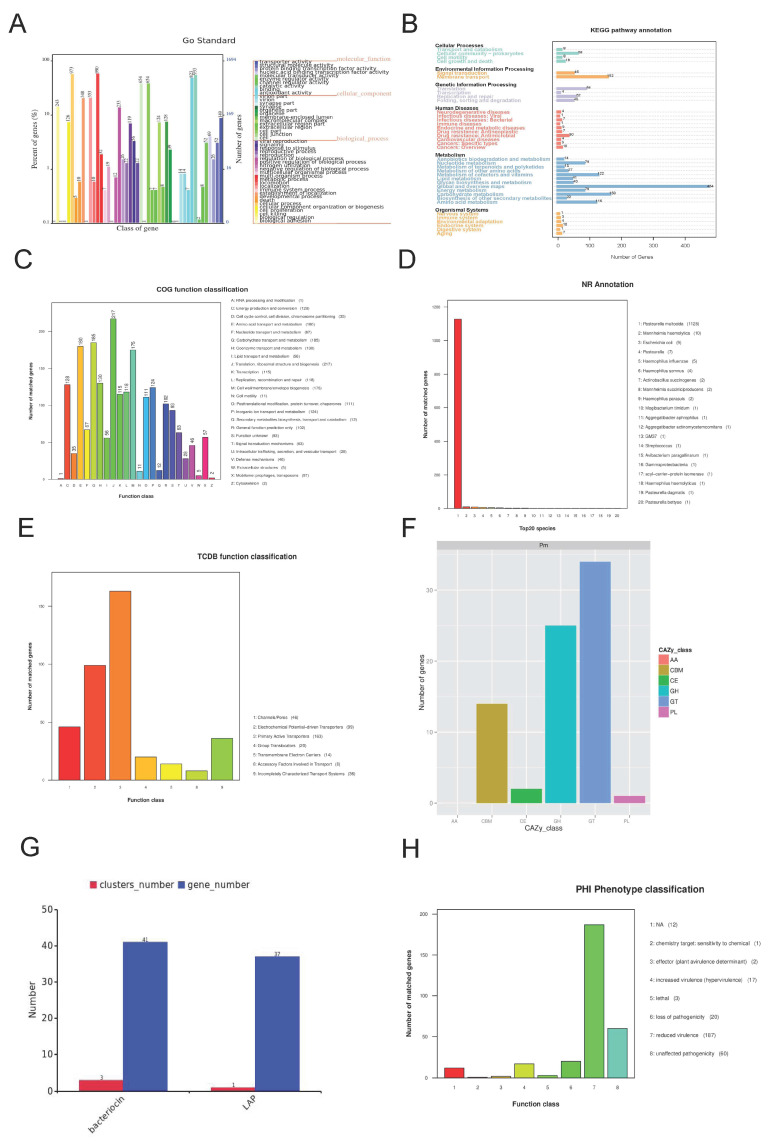
Provides comprehensive insights into the functional annotation of *P. multocida* SHZ01. (**A**) GO Functional Classification Diagram: This diagram illustrates genes’ Gene Ontology (GO) functional classification. The horizontal axis depicts the GO functional categories, the right vertical axis indicates the number of genes annotated for each category, and the left vertical axis represents the percentage of genes in each category relative to all coding genes. (**B**) KEGG Metabolic Pathway Classification Diagram: The bar chart displays the classification of genes into different metabolic pathways according to the Kyoto Encyclopedia of Genes and Genomes (KEGG). Numbers on the bars denote the count of genes annotated for each pathway. The other coordinate axis provides the code for each level 1 functional class in the KEGG database. (**C**) COG Functional Classification Diagram: This diagram represents the functional classification of genes based on the Clusters of Orthologous Groups (COG) database. The horizontal axis indicates COG functional types, and the vertical axis shows the number of genes annotated for each type. (**D**) NR Functional Classification Diagram: In this diagram, the horizontal axis represents species ID, and the vertical axis indicates the number of genes annotated for each species in the Non-Redundant Protein Database (NR). (**E**) TCDB Function Classification Diagram: The horizontal axis represents the TCDB primary classification type, and the vertical axis represents the number of genes on the annotation. (**F**) CAZy Functional Classification and Corresponding Gene Quantity Statistical Chart: The horizontal axis represents the classification type of the CAZy database, from left to right—Auxiliary Activities (AAs); cCarbohydrate-Related Modules (CBMs); Carbohydrate Esterases (CEs); Glycoside Hydrolases (GHs); Glycosyl Transferases (GTs); Polysaccharide Lyases (PLs). The vertical axis represents the number of genes annotated. (**G**) Statistical Diagram of Gene Clusters and Corresponding Gene Numbers: The horizontal axis represents Gene cluster name, and the vertical axis represents the number of genes contained in gene clusters. (**H**) Distribution Map of Pathogen PHI Phenotypic Mutation Types: The horizontal axis represents the type of phenotype mutation, and the vertical axis represents the number of genes on the annotation.

In the Gene Ontology (GO) database, a total of 1693 genes within the *P. multocida* SHZ01 strain were successfully annotated. The GO analysis categorized these genes into three main classes: molecular functions, biological processes, and cellular components. Across these categories, 47 subcategories emerged, with cellular components, molecular functions, and biological processes further subdivided into 13, 10, and 24 categories, respectively. Notably, the most enriched genes were identified in specific categories, highlighting key functional aspects. In biological processes, the metabolic process pathway stood out, with 990 genes, while catalytic activity in molecular function exhibited 933 genes. Within cellular components, both the cell class and cell part class were prominent, featuring 654 genes (Figure 7A). This detailed categorization provides insights into the diverse functional roles of genes within the *P. multocida* SHZ01 strain, emphasizing key pathways and activities.

#### 3.4.2. KEGG Annotation Results

In the KEGG database, a total of 2226 genes within the *P. multocida* SHZ01 strain were annotated. The KEGG database encompasses diverse categories such as metabolic pathways (KEGG PATHWAY), drugs (KEGG DRUG), diseases (KEGG DIS-EASE), functional models (KEGG MODULE), gene sequences (KEGG GENES), and genomes (KEGG GENOME). Through a KEGG analysis of the SHZ01 strain, it is evident that the global and overview maps pathways, along with the carbohydrate metabolism pathway in the metabolic pathways category, exhibit the highest number of enriched genes. Specifically, the global and overview maps pathways have 464 and 159 enriched genes, respectively (Figure 7B, Table S6). A total of 42 genes were enriched in the quorum sensing pathway (Figure S3).

#### 3.4.3. COG Annotation Results

The COG annotation results for the *P. multocida* SHZ01 strain reveal a total of 1881 annotated genes, categorized into 24 functional groups based on their functions. The annotation results show that the translation, ribosomal structure, and biogenesis pathways, along with the carbohydrate transport and metabolism pathways, are the most gene-rich. Specifically, the translation pathway encompasses 217 genes, and the carbohydrate transport and metabolism pathways include 185 genes (Figure 7C). This observation aligns with the findings from the KEGG analysis, reinforcing the significance of these pathways in the life processes of bacteria. The abundance of genes associated with translation and carbohydrate metabolism underscores their crucial roles in the functional repertoire of *P. multocida* SHZ01.

#### 3.4.4. NR, Pfam, and Swiss-Prot Annotation

In the non-redundant protein database (NR), the predicted protein sequence of *P. multocida* SHZ01 was compared, resulting in the annotation of 2332 genes. Among these, *P. multocida* itself had the largest number of annotated genes, with 1128 genes (Figure 7D). Moving on to the Pfam database, which identifies protein functions by recognizing protein domains, a total of 1693 genes from the *P. multocida* SHZ01 strain are annotated in this database. Additionally, the Swiss-Prot database, providing comprehensive protein annotation results, annotated 1715 genes from the *P. multocida* SHZ01 strain. These annotations, spanning various databases, contribute to a comprehensive understanding of the functional aspects of the *P. multocida* SHZ01 genome.

#### 3.4.5. TCDB Annotation

The Transporter Classification Database (TCDB), specializing in membrane transport proteins, annotated 386 genes from the *P. multocida* SHZ01 strain. Notably, among these annotations, the categories of Primary Active Transporters and Electrochemical potential-driven Transporters stand out, boasting 163 and 99 annotated genes, respectively (Figure 7E). This detailed classification sheds light on the membrane transport functionalities within the *P. multocida* SHZ01 genome.

#### 3.4.6. Carbohydrate-Active Enzymes (CAZy) Annotation

The Carbohydrate Active Enzyme (CAZy) database is a collection of enzymes related to the degradation, modification, and biosynthesis of carbohydrates. It is mainly divided into five categories: Glycoside Hydrolases (GHs), Glycosyl Transferases (GTs), Polysaccharide Lyases (PLs), Carbohydrate Esterases (CEs), and Oxidoreductases (Auxiliary Activities, AAs). In the *P. multocida* SHZ01 strain, a total of 74 genes were annotated in the CAZy database. Among these genes, glycosyltransferase had the largest number of annotations, with 34 genes (Figure 7F).

### 3.5. Analysis of Secretion Systems, Secondary Metabolism Gene Clusters

The predicted effectors for the *P. multocida* SHZ01 strain include proteins involved in secretion systems and gene clusters related to the secondary metabolism. Pathogens engage in the host’s immune response through the secretion system. Secretion system proteins were extracted from the results of protein function annotation for further analysis. The type III secretion system (T3SS) is a virulence factor unique to Gram-negative bacteria that enables the secretion of effector molecules into the host cytoplasm to evade the immune system. The *P. multocida* SHZ01 strain was predicted to have a total of 2418 secretion system proteins, out of which 80 were predicted to be T3SS effector proteins. A lot of hypothetical proteins were classified as T3SS effector proteins. Two virulence genes, namely *Pgi* and *hemX*, were also classified as T3SS effector proteins, which are involved in the carbohydrate metabolism and heme biosynthesis. Four iron transport system-associated genes, *sitB*, *ABC.FEV.A,* and *fdoH*, *fdsB,* were classified as T3SS effector proteins. In the analysis of T3SS, there were three annotated genes—*wza* and *gfcE*, associated with biofilm formation in *Escherichia coli* and *Vibrio cholerae*, and *secB*, involved in the quorum sensing pathway. Furthermore, we identified an SAM-dependent methyltransferase, derived from the *P. multocida* ATCC 43137 strain, annotated in the KEGG database. This methyltransferase is known to catalyze methylation reactions that are crucial for various physiological processes, including biosynthesis, signal transduction, and protein repair, with substrate specificity determining the diverse functional outcomes [28].

Secondary metabolites are substances that microorganisms synthesize from primary metabolites as precursors during a specific growth period. These metabolites do not have a clear function in the activities of microorganisms and are not necessary for growth and reproduction (Table S7). The *P. multocida* SHZ01 strain has two predicted secondary metabolism gene clusters, namely bacteriocin and leucine arylamidase (LAP). Among them, bacteriocin has a large number of annotated genes, with 41 genes (Figure 7G).

### 3.6. Analysis of Virulence Genes and Drug Resistance Genes

The PHI database is a pathogen–host interaction database. The *P. multocida* SHZ01 strain has 275 genes annotated in this database, with 7 mutant phenotypes. Among these, 14 genes have 2 or more phenotypes (Figure 7H). The *P. multocida* SHZ01 strain has a total of 82 virulence genes annotated in the VFDB database, which can be divided into adherence, invasion, immune modulation, motility, antiphagocytosis, and iron uptake according to their functional classification. Most of these genes belong to lipooligosaccharides with immunomodulatory functions. There are also many genes belonging to capsular polysaccharides with anti-phagocytic function (Table 3). The ARBD database can provide information on the resistance-related genes of species and the antibiotics that they are resistant to. The CARD database integrates information such as sequences, antibiotic resistance, mechanisms of action, and associations between antibiotic resistance organisms (AROs). The *P. multocida* SHZ01 strain has 54 types of resistance genes annotated in the CARD database, including fluoroquinolones, β-lactams, glycopeptides, aminoglycosides, amides, streptomycin, tetracyclines, polymyxins, etc. (Table S8).

## 4. Discussion

In veterinary clinical practice, there are many reports of co-infection between Mo and *P. multocida*, and the double-positive rate of *P. multocida* and Mo antibodies is relatively high in sheep populations in Xinjiang and other regions. Scholars have found that co-infection of Mo and *P. multocida,* causing pneumonia, is the main factor leading to the death of argali sheep. The infection of argali sheep with Mo alone does not cause fatal pneumonia. Mo infection likely suppresses the lung immunity of sheep, leading to mixed infections from bacteria such as *P. multocida* [6,7,8,9]. Mo infection in argali hybrid sheep and subsequent secondary infection with *P. multocida* represent significant challenges for the development of the argali hybrid sheep industry. The secondary *P. multocida* infection induces symptoms such as fever, cough, dyspnea, and pulmonary consolidation in argali hybrid sheep, posing a substantial threat to the sheep industry. In this study, we successfully isolated and identified *P. multocida* from the lungs of argali hybrid sheep infected with Mo. The mouse pathogenicity experiment underscored the high pathogenicity of the *P. multocida* SHZ01 strain. The symptoms of mice infected with *P. multocida* SHZ01 strain may be consistent with pneumonia, with pathological features such as hemorrhage, edema, and fibrinous exudation in the lungs. This suggests that the SHZ01 strain may exacerbate Mo infection in sheep.

A whole genome characterization of this strain of *P. multocida*, named SHZ01, was conducted. In the NCBI gene database, there are very few whole genome sequencing data of *P. multocida* originating from sheep, and most are from bovine and avian species [29]. *P. multocida* originating from argali hybrid sheep seems to have not been sequenced. Employing whole genome second-generation sequencing, we conducted a comprehensive analysis, predicting two gene islands and identifying 80 T3SS effector proteins in the *P. multocida* SHZ01 strain. The Type III Secretion System (T3SS) is a distinctive virulence factor found in Gram-negative bacteria, facilitating the secretion of effector molecules into the host cytoplasm as a strategy to evade the immune system [30]. Notably, the Dot/Icm Type IV secretion system effector has been implicated in the survival and replication of *Legionella pneumophila* within macrophages, particularly during its interaction with human alveolar macrophages [31]. This discovery suggests that the *P. multocida* SHZ01 strain may employ a similar secretion system to invade alveolar macrophages, ensuring persistence and inducing cellular disease. Quorum sensing pathways are involved in bacterial biofilm formation. Additionally, the genes of *wza*, *gfcE,* and *secB* may be involved in the process of biofilm formation in *P. multocida*. Interestingly, the *P. multocida* SHZ01 strain exhibits a quorum sensing pathway (Figure S3, Table S6), as annotated in the KEGG database, distinguishing it from the porcine *P. multocida* NIVEDIPm17 strain [26]. Additionally, our analysis revealed the annotation of the *htpB* gene in the Legionellosis pathway within the KEGG database. *HtpB* encodes a chaperone protein known to modify eukaryotic cell signaling pathways, facilitating the entry of bacteria into the mammalian cytoplasm. This capability enhances bacterial replication within host cells [32]. The identification of the *htpB* gene in the Legionellosis pathway strongly suggests that the *P. multocida* SHZ01 strain may employ this gene to communicate with host cells. This interaction could play a crucial role in the pathogenesis of the infection.

Additionally, we identified 82 virulence genes, encompassing key virulence factors such as lipopolysaccharide (LPS), lipooligosaccharides (LOS), capsular polysaccharides, and iron uptake proteins. Noteworthy secondary virulence genes include *IlpA*, *flmH*, and *algU*. Extensive research has underscored the significance of virulence genes in *P. multocida* infections [33]. Different virulence genes are associated with specific diseases and host species [29]. For instance, the *toxA* gene, encoding PMT, serves as the primary virulence factor responsible for atrophic rhinitis in pigs [34]. The filamentous hemagglutinin surface adhesin functions as a major virulence factor causing pneumonia in birds and cattle [35]. Moreover, the capsular polysaccharide stands out as the main virulence factor behind avian cholera and hemorrhagic sepsis [36]. LOS, a key component of LPS, along with the capsule, plays an essential role in *P. multocida* pathogenesis [37]. In our analysis, we annotated two genes encoding enzymes, *rfaD* and *rfaE*, that are crucial for LOS synthesis in the *P. multocida* SHZ01 strain. The deletion of these genes could result in shorter sugar chains of bacterial LOS. LOS phosphocholine (ChoP) has the potential to influence invasion and trigger inflammatory signaling by interacting with PAF receptors. The phase-variable expression of LPS biosynthetic genes facilitates the evasion of antigen-specific host immune defenses, enabling colonization in diverse host microenvironments [38]. The capsule of *P. multocida* serotype A is a critical virulence factor [29], and the *P. multocida* SHZ01 strain contained 14 capsule-related genes, which are able to help the *P. multocida* adhere to and enter host cells as well as resist macrophage phagocytosis. Previous research has highlighted the significance of iron uptake proteins in the survival and pathogenesis of *P. multocida* [39]. In gram-negative bacteria, the iron regulatory system is typically governed by a Ferric uptake regulator (Fur) [40]. However, certain bacteria, including *Escherichia coli* and *Campylobacter jejuni*, employ homologues of Fur such as the ChuA outer membrane protein. These proteins facilitate the utilization of heme- and hemoglobin as an effective iron acquisition strategy during infection [41,42]. Our annotation identified the virulence gene *fur*, along with its homolog ChuV, which shares a similar structure to ChuA, in the *P. multocida* SHZ01 strain. This suggests that the *P. multocida* SHZ01 strain has the ability to absorb iron both through Fur and ChuV from the host. Many iron transport system-related genes and heme biosynthesis-related genes originated in the gene island and T3SS, indicating that *P. multocida* SHZ01 is likely to cause disease through iron acquisition. The presence of these virulence factors encoding genes could potentially enhance bacterial virulence during infection [43]. 

The *P. multocida* SHZ01 strain exhibits two significant virulence factors: the *IlpA* gene and polar flagella. The *IlpA* gene encodes immunogenic lipoprotein A, serving dual roles as an adhesin and an immunostimulant. It induces the production of pro-inflammatory cytokines in human monocytes through Toll-like receptor 2 (TLR2), triggering an inflammatory response [44]. Polar flagella are crucial for bacterial motility, adhesion, and invasion, with the glycosylation of flagellin potentially contributing to proinflammatory responses [45,46]. Additionally, the *P. multocida* SHZ01 strain harbors the *algU* gene, which is responsible for encoding alginate, a factor facilitating biofilm formation [47]. In *Pseudomonas aeruginosa*, alginate plays a role in bacterial persistence in the lungs of individuals with cystic fibrosis (CF) by acting as an adhesin that prevents expulsion and forming a mucus layer that impedes phagocyte clearance [48]. Screening for virulence genes further reveals the presence of additional secondary virulence genes in the *P. multocida* SHZ01 strain, contributing to bacterial invasion and encompassing functions related to immune evasion, environmental adaptation, and essential nutrient uptake.

The analysis of the Comprehensive Antibiotic Research Database (CARD) demonstrates the existence of multiple resistance genes in the *P. multocida* SHZ01 strain. These genes confer resistance to fluoroquinolones, β-lactams, glycopeptides, aminoglycosides, amides, streptomycin, tetracycline, and polymyxin. Tetracycline and glycopeptide resistance genes are particularly prevalent, aligning with previous findings that tetracycline resistance is widespread among *P. multocida* isolates across different species. Notably, glycopeptide resistance genes have been identified in *P. multocida* strains derived from various hosts, including the porcine-derived *P. multocida* strain NIVEDIPm17 [26], the Australian cattle-derived *P. multocida* 17BRD-035 strain [49], and the duck-derived *P. multocida* strain PMWSG-4 [33] The widespread occurrence of tetracycline and glycopeptide resistance genes may be attributed to the common use of these antibiotics in production practices to address diseases caused by *P. multocida*.

## 5. Conclusions

In this study, a serotype *P. multocida* strain was isolated from the lungs of a Mo-infected argali hybrid sheep in China. Pathogenicity experiments conducted in mice revealed that *P. multocida* infection induced pathological features such as pulmonary hemorrhage, edema, and the disruption of the splenic cord structure. Subsequent genomic analysis involved sequencing the draft genome of *P. multocida* SHZ01, aiming to unravel the genetic factors contributing to its adaptations and pathogenesis during infection.

The genomic analysis identified several key genes that may play crucial roles in the bacterium’s adaptability and pathogenicity. For instance, the genes *rfaD* and *rfaE* were found to be involved in lipopolysaccharide (LPS) synthesis, a critical component in the outer membrane of gram-negative bacteria. Another notable gene, *IlpA*, was identified, which is implicated in both adhesion and inflammation processes. The iron transport system and heme biosynthesis-related genes are likely to be factors in the pathogenesis of *P. multocida*. Additionally, the presence of multiple antibiotic resistance genes and virulence factor-encoding genes suggests a potential increase in bacterial antibiotic resistance and virulence during infection.

This comprehensive genomic analysis provides valuable insights into the genetic basis of *P. multocida* pathogenesis. In the next phase of the study, the pathogenic mechanism will be further investigated through co-infection experiments involving Mo and *P. multocida* in hybrid sheep. This research will contribute to a deeper understanding of the molecular interactions between these pathogens and shed light on the complexities of disease progression in hybrid sheep co-infected with Mo and *P. multocida.*

## Figures and Tables

**Table 1:** General characteristics of the genome of *P. multocida* SHZ01 strain.

Item	Number	Item	Number
Genome Size (bp)	2,378,508	Genomics Islands Number	2
Gene Number	2418	Genomics Islands Total Length (bp)	12,256
Combined Gene Length (bp)	2,051,916	Average Genomics Islands Length (bp)	6128
Gene Average Length (bp)	849	Prophage Number	19
Total Length of Intergenic Region (bp)	326,592	Prophage Total Length (bp)	137,621
GC Content (%)	40.89	Prophage Average Length (bp)	7243.21
Intergenic Region GC Content (%)	35.72	rRNA Number	13
Gene Length/of Genome (%)	86.27	tRNA Number	58
Intergenic region Length/Genome (%)	13.73	sRNA Number	8
Tandem Repeat Number	80	CRISPR Number	8
Tandem Repeat (bp)	5–144	CRISPR Length (bp)	1199
Tandem Repeat Total Length (bp)	4511	CRISPR Average Length (bp)	149.875
Tandem Repeat Total Length/Genome (%)	0.1897	Microsatellite DNA Number	4
Minisatellite DNA Number	64		

**Table 2:** Collaborating strain information.

Strain (GenBank Accession No.)	Host	Year	Country	Serogroup
*Pasteurella multocida* 40540 (CP097796.1)	turkey	2022	Denmark	A:12
*Pasteurella multocida* 29135 (CP097797.1)	turkey	2022	Denmark	A:10, 12
*Pasteurella multocida* LXSS001 (CP119523.1)	rabbit	2022	China	/
*Pasteurella multocida* 33011 (CP097612.1)	avian	2022	USA	/
*Pasteurella multocida* PF17 (CP112895.1)	rabbit	2021	China	F
*Pasteurella multocida* IMT47951 (CP087380.1)	bovine	2019	Germany	/
*Pasteurella multocida* HN02 (CP037865.1)	sheep	2018	China	/
*Pasteurella multocida* RCAD0730 (CP059704.1)	duck	2018	China	/
*Pasteurella multocida* HN07 (CP007040.1)	pig	2017	China	F
*Pasteurella multocida* Pm1 (AP025519.1)	bovine	2017	Japan	/
*Pasteurella multocida* 17BRD-035 (CP082272.1)	bovine	2017	Australia	/
*Pasteurella multocida* 3480 (CP001409.1)	pig	2014	USA	A
*Pasteurella multocida* USDA-ARS-USMARC-60712 (CP015566.1)	bovine	2014	USA	/
*Pasteurella multocida* CQ2 (CP033599.1)	bovine	2013	China	A
*Pasteurella multocida* HN06 (NC_017027.1)	pig	2003	China	D

**Table 3:** Virulence gene classification.

Function	Virulence Factors	Related Genes
Adherence	Polar flagella	*flmH*
	IlpA	*IlpA*
	Flagella	*fleR/flrC*
	Alginate	*algU*
	type IV pili	*vfr*, *hofC*, *ptfA*, *comE/pilQ*
	Hsp60	*htpB*
	P5 protein	*ompP5*
	EF-Tu	*Fphi_1039*
	Autoinducer-2	*luxS*
	ClpP	*clpP*
Invasion	LPS	*OOM_1046*, *fabZ*, *acpXL*, *kdtB*
	Polar flagella	*flmH*
	LOS	*rfaD*, *rfaE*
	Chu	*chuV*
	Capsule	*lipA*
Immune modulation	LOS	*wecA*, *msbB*, *lpxB*, *lpxA*, *lpxD*, *rfaD*, *rffG*, *yhxB/manB*, *lpxC*, *gmhA/lpcA*, *lpxH*, *lsgA*, *lsgB*, *lsgD*, *lsgE*, *lsgF*, *kpsF*, *kdsA*, *lex2B*, *lic2A*, *lgtA*, *lgtC*, *orfM*, *htrB*, *rfaE*, *msbA*, *lpxK*, *kdsB*, *galU*, *waaQ*, *msbA*, *opsX/rfaC*, *kdkA*, *kdtA*, *lgtF*, *CFF8240_1412*, *galE*, *opsX/rfaC*, *rfaF*
Motility	Polar flagella	*flmH*
Antiphagocytosis	Capsule	*KOX_25165*, *uppS*, *oppF*, *BJAB07104_00090*, *bexA*, *ctrC*, *bexC*, *bexD*, *ugd*, *oppF*, *hscB*, *lipA*, *gnd*, *Fphi_1467*
Iron uptake	Fur	*fur*
	Chu	*chuV*
Biofilm formation	Flagella	*fleR/flrC*
	Alginate	*algU*

## Data Availability

The raw data supporting the conclusions of this article will be made available by the authors on request.

## Supplementary Materials

The following supporting information can be downloaded at: https://www.mdpi.com/article/10.3390/microorganisms12061072/s1, Figure S1: Identification of *P. multocida* SHZ01 strain. (A) Identification of *P. multocida* isolated from nasal swabs collected from 4 sheep; 1–4, 6–9, 11–13 and 14–16 were bacteria isolated from 4 sheep respectively. (B)After SHZ01 strain infected mice, some lung tissues were taken to identify *P. multocida;* Figure S2: Mice infected with the *P. multocida* SHZ01 strain developed small white spots in their livers. The white spotes are labeled with white arrow; Figure S3: Quorum sensing pathway picture; Table S1. Primer sequences for identification of *P. multocida* SHZ01 strain; Table S2. PCR reaction system of bacterial identification; Table S3. PCR reaction program of bacterial species identification; Table S4. PCR reaction program of capsular serological identification; Table S5. Gene annotation ratio statistics; Table S6. KEGG maps pathways annotations; Table S7.Scattered repeat sequence result statistics; Table S8. Summary table of drug resistance function annotations.
