# Supplementary material for: Pathogenicity and Genomic Characteristics Analysis of Pasteurella multocida Serotype A Isolated from Argali Hybrid Sheep
Source: Microorganisms. 2024 May 25;12(6):1072. doi: 10.3390/microorganisms12061072 (PMC11205410; doi:10.3390/microorganisms12061072)

Figure S2. Mice infected with the *P. multocida* SHZ01 strain developed small white spots in their livers. The white spotes are labeled with white arrow.

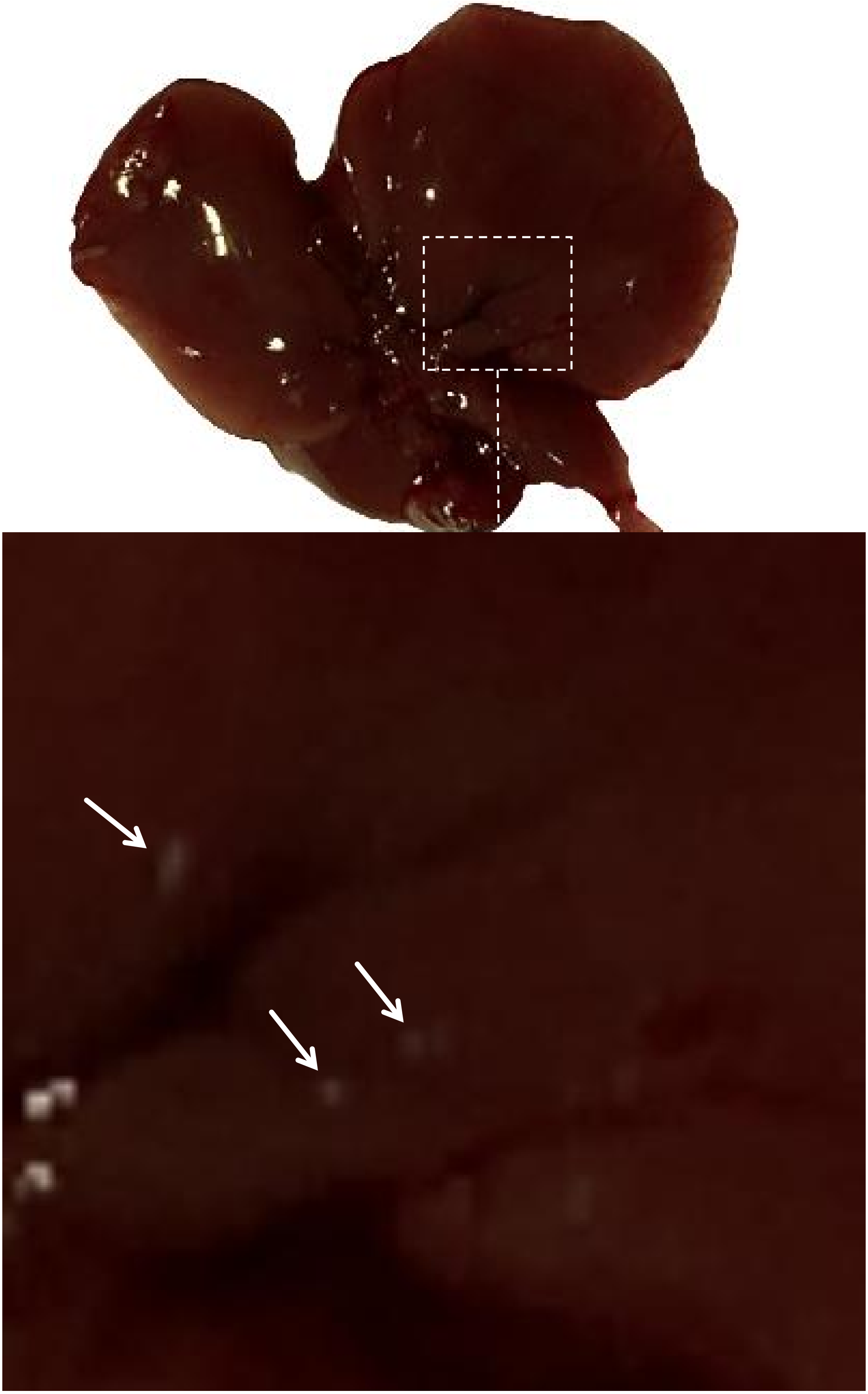

Supplement: Supplementary file 1 [file microorganisms-12-01072-s001.zip › Figure S2.pdf]
