# Supplementary material for: Pathogenicity and Genomic Characteristics Analysis of Pasteurella multocida Serotype A Isolated from Argali Hybrid Sheep
Source: Microorganisms. 2024 May 25;12(6):1072. doi: 10.3390/microorganisms12061072 (PMC11205410; doi:10.3390/microorganisms12061072)
Supplement: Supplementary file 1 [file microorganisms-12-01072-s001.zip › Supplemental figs.pdf]

Pathogenicity and Genomic Characteristics Analysis of *Pasteurella Multocida*

Serotype A Isolated from Argali Hybrid Sheep

Xinyan Cao1, Gang Wang2, Lanying Gu1, Zhiyu Gao1, Wenyu Fan1, Qinchuan Zhang1, Jinliang Sheng1, Yanbing Zhang1,* and Yanming Sun1,*

Figure S1 Identification of *Pasteurella multocida* strain SHZ01.(A)Identification of *Pasteurella multocida* isolated from nasal swabs collected from 4 sheep;1-4, 6-9, 11-13 and 14-16 were bacteria isolated from 4 sheep respectively. (B)After SHZ01 strain infected mice, some lung tissues were taken to identify *Pasteurella multocida*

A

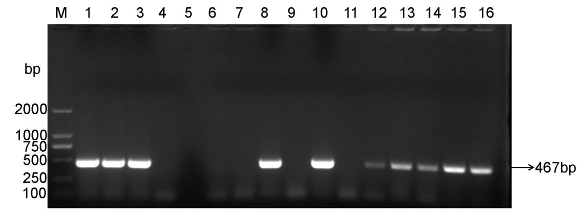

B

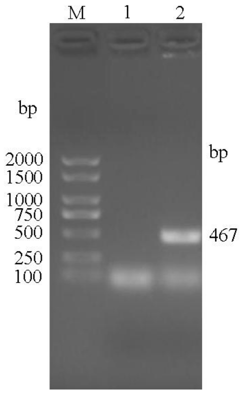

Figure S3 Quorum sensing pathway picture

A

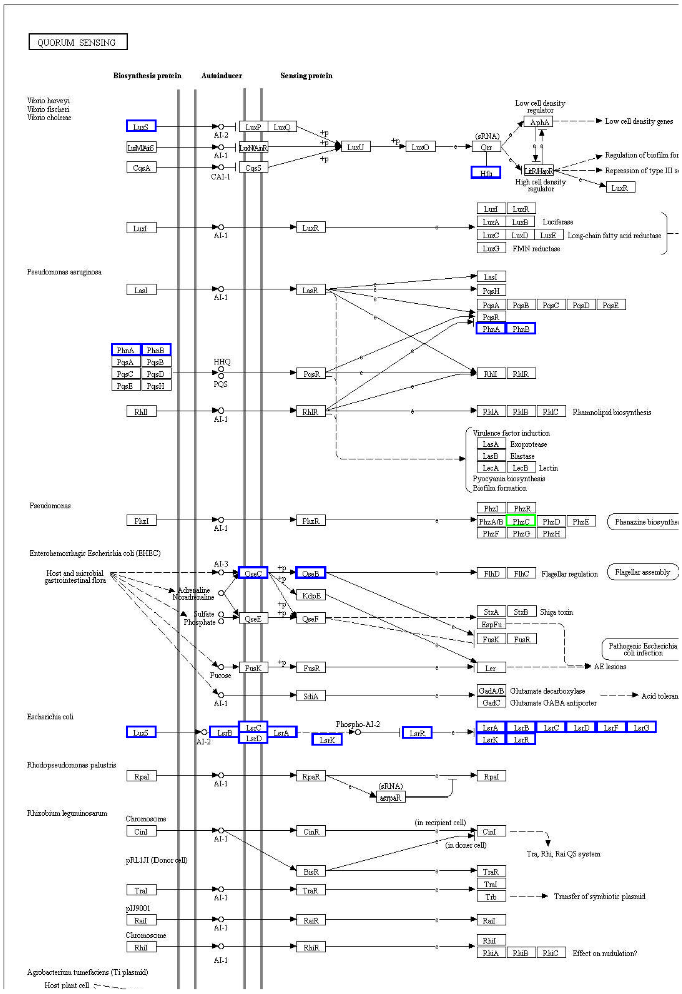
QUORUM SENSING
Biosynthesis protein
Autoinducer
Sensing protein
Vibrio harveyi
Vibrio fischeri
Vibrio cholerae
LuxS
AI-2
LuxP
LuxQ
LuxM/AinS
AI-1
LuxN/AinR
CqsA
CAI-1
CqsS
LuxU
LuxO
(sRNA)
Qrr
Hfq
Low cell density regulator
AphA
Low cell density genes
LuxR/HapR
High cell density regulator
Regulation of biofilm for
Repression of type III s
LuxR
LuxI
LuxR
Luciferase
Long-chain fatty acid reductase
FMN reductase
Pseudomonas aeruginosa
LasI
LasR
PqsH
PqsA
PqsB
PqsC
PqsD
PqsE
PqsR
PhnA
PhnB
HHQ
PQS
RhlI
RhlR
RhlA
RhlB
RhlC
Rhamnolipid biosynthesis
Virulence factor induction
LasA
Exoprotease
LasB
Elastase
LecA
LecB
Lectin
Pyocyanin biosynthesis
Biofilm formation
Pseudomonas
PhzI
PhzR
PhzA/B
PhzC
PhzD
PhzE
PhzF
PhzG
PhzH
Phenazine biosynthe
Enterohemorrhagic Escherichia coli (EHEC)
Host and microbial gastrointestinal flora
AI-3
QseC
QseB
Adrenaline
Noradrenaline
KdpE
Sulfate
Phosphate
QseE
QseF
FlhD
FlhC
Flagellar regulation
Flagellar assembly
StxA
StxB
Shiga toxin
EspFu
FusK
FusR
Pathogenic Escherichia coli infection
Ler
AE lesions
Fucose
SdiA
GadA/B
Glutamate decarboxylase
GadC
Glutamate GABA antiporter
Acid toleran
Escherichia coli
LsrB
LsrC
LsrD
LsrA
LsrK
Phospho-AI-2
LsrR
LsrF
LsrG
Rhodopseudomonas palustris
RpaI
RpaR
(sRNA)
asrpaR
Rhizobium leguminosarum
Chromosome
CinI
CinR
(in recipient cell)
(in donor cell)
Tra, Rhi, Rai QS system
pRL1JI (Donor cell)
BisR
TraR
TraI
Trb
Transfer of symbiotic plasmid
pIJ9001
RaiI
RaiR
RhiI
RhiR
RhiA
RhiB
RhiC
Effect on nodulation?
Agrobacterium tumefaciens (Ti plasmid)
Host plant cell

B

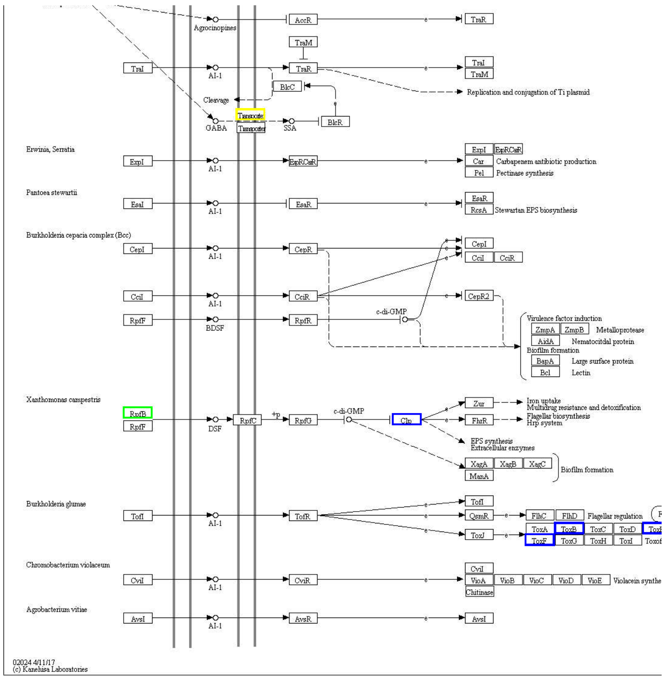
Agrocinopines
AccR
TraR
TraM
TraI
AI-1
BlcC
Cleavage
Transporter
GABA
SSA
BlcR
Replication and conjugation of Ti plasmid
Erwinia, Serratia
ExpI
ExpR/CarR
Car
Carbapenem antibiotic production
Pel
Pectinase synthesis
Pantoea stewartii
EsaI
EsaR
RcsA
Stewartan EPS biosynthesis
Burkholderia cepacia complex (Bcc)
CepI
CepR
CciI
CciR
CepR2
RpfF
BDSF
RpfR
c-di-GMP
Virulence factor induction
ZmpA
ZmpB
Metalloprotease
AidA
Nematocidal protein
Biofilm formation
BapA
Large surface protein
Bcl
Lectin
Xanthomonas campestris
RpfB
RpfF
DSF
RpfC
RpfG
Clp
Zur
Iron uptake
Multidrug resistance and detoxification
FhrR
Flagellar biosynthesis
Hrp system
EPS synthesis
Extracellular enzymes
XagA
XagB
XagC
ManA
Biofilm formation
Burkholderia glumae
TofI
TofR
QsmR
ToxJ
FlhC
FlhD
Flagellar regulation
ToxA
ToxB
ToxC
ToxD
ToxF
ToxG
ToxH
ToxI
Chromobacterium violaceum
CviI
CviR
VioA
VioB
VioC
VioD
VioE
Violacein synthe
Chitinase
Agrobacterium vitiae
AvsI
AvsR
02024 4/11/17
(c) Kanehisa Laboratories
