# Supplementary material for: Pathogenicity and Genomic Characteristics Analysis of Pasteurella multocida Serotype A Isolated from Argali Hybrid Sheep
Source: Microorganisms. 2024 May 25;12(6):1072. doi: 10.3390/microorganisms12061072 (PMC11205410; doi:10.3390/microorganisms12061072)
Supplement: Supplementary file 1 [file microorganisms-12-01072-s001.zip › Supplementary tables.pdf]

Pathogenicity and Genomic Characteristics Analysis of Pasteurella Multocida Serotype A Isolated from Argali Hybrid Sheep

Xinyan Cao, Gang Wang, Lanying Gu, Zhiyu Gao, Wenyu Fang, Qinchuan Zhang, Jinliang Sheng, Yanbing Zhang*, Yanming Sun*

Table S1. Primer sequences for identification of *Pasteurella multocida* strain SHZ01

Serogroup	Gene	Primers name	Primers sequence	Amplimer size(bp)
All	KMT1	KMT1T7	5′-ATCCGCTATTTACCCAGTGG- 3′	467
		KMT1SP6	5′-GCTGTAAACGAACTCGCCAC- 3′	
A	hyaD-hyaC	CAPA-FWD	5′-TGCCAAAATCGCAGTCAG- 3′	1044
		CAPA-REV	5′-TTGCCATCATTGTCAGTG- 3′	
B	bcbD	CAPB-FWD	5′-CATTTATCCAAGCTCCACC- 3′	760
		CAPB-REV	5′-GCCCGAGAGTTTCAATCC- 3′	
D	dcbF	CAPD-FWD	5′-TTACAAAAGAAAGACTAGGAGCCC- 3′	657
		CAPD-REV	5′-CATCTACCCACTCAACCATATCAG- 3′	
E	ecbJ	CAPE-FWD	5′-TCCGCAGAAAATTATTGACTC- 3′	511
		CAPE-REV	5′-GCTTGCTGCTTGATTTTGTC- 3′	
F	fcbD	CAPF-FWD	5′-AATCGGAGAACGCAGAAATCAG- 3′	851
		CAPF-REV	5′-TTCCGCCGTCAATTACTCTG- 3′	

Table S2. PCR reaction system of bacterial identification

Reaction component	Volume（μL）
BioGold 2×SuperPCR Master Mix	10
Forward primer	1
Reverse primer	1

Bacterial DNA	1
ddH_2O	7
Total	20

Table S3. PCR reaction program of bacterial species identification

Cycle	Temperature(℃)	Time
	95	5 min
	95	20 second
30	57	30 second
	72	45 second
	72	10 min

Table S4. PCR reaction program of capsular serological identification

Cycle	Temperature(℃)	Time
	95	5 min
	95	30 second
30	55	30 second
	72	30 second
	72	5 min

Table S5. Gene annotation ratio statistics

Annotation database	Number of genes	Annotation ratio (%)
NR	2,332	18.43
SwissProt	1,715	13.55
KEGG	2,226	17.59

COG	1,881	14.86
TCDB	386	3.05
GO	1,693	13.38
PHI	302	2.39
VFDB	164	1.30
ARDB	0	0
CARD	66	0.52
Secretory_Protein	123	0.97
T3SS	1	0.00
CAZy	74	0.58
Pfam	1,693	13.38
Total number of genes	12656	100

Table S7. Scattered repeat sequence result statistics

Sample ID	Type	Number(#)	Total Length(bp)	In Genome(%)	Average length(bp)
Pm	LTR	71	5,511	0.2317	79
Pm	DNA	27	1,449	0.0609	54
Pm	LINE	21	1,310	0.0551	62
Pm	SINE	7	480	0.0202	69
Pm	RC	3	167	0.007	56
Pm	Unknown	0	0	0	0
Pm	Total	129	8,795	0.3698	70

Table S8. Summary table of drug resistance function annotations

ARO_name	Antibiotic resistance
tet34, tetB(P), tetS, tetT, tet32, tetM, tetO, otr(A), tet36, tet44, tetB(P), tetQ, tet44, tetT, tetW, tetS, tetM, tetO, otr(A), tet36, tet32	Tetracycline resistance
mecC, mecB, mecA, Neisseria meningititis PBP2 conferring resistance to beta-lactam, NmcR	Beta-lactam resistance
Mfd, gyrA, parC	Fluoroquinolone resistance
murA	Fosfomycin resistance
fabI	Isoniazid resistance
fabI	Triclosan resistance
arlR, vanRA, vanRE, vanRF, vanRI, vanRM, CpxR, vanRM, vanRI, vanRC, vanRD, vanRE, vanRF, vanRG, mtrA, kdpE, smeR, vanRN, vanHB, vanHA, vanHF, vanHD, vanHO, vanHM, vanTN, vanTrL, vanTE, vanTC, vanTG, vanA, vanC, vanB, vanE, vanD, vanG, vanF, vanM, vanL, vanO, vanN,	Glycopeptide resistance
ileS	Mupirocin resistance
clbC, clbB, clbA, cipA, cfrA, cfrC	Multiple resistances
basS,PmrC, MCR-1, MCR-2, MCR-3, PmrE	Polymyxin resistance
sul3	Sulfonamide resistance
EF-Tu	Elfamycin resistance
vatC, vatB, vatA, vatF, vatE, vatD, VatI, vatH	Streptogramin resistance
parY	Aminocoumarin resistance
